# PI3K pathway mutation predicts an activated immune microenvironment and better immunotherapeutic efficacy in head and neck squamous cell carcinoma

**DOI:** 10.1186/s12957-023-02938-6

**Published:** 2023-03-02

**Authors:** Libo Wang, Kejun Chen, Siyuan Weng, Hui Xu, Yuqing Ren, Quan Cheng, Peng Luo, Jian Zhang, Zaoqu Liu, Xinwei Han

**Affiliations:** 1 Department of Interventional Radiology, The First Affiliated Hospital of Zhengzhou University, Zhengzhou, 450052 Henan People’s Republic of China; 2 Department of Hepatobiliary and Pancreatic Surgery, The First Affiliated Hospital of Zhengzhou University, Zhengzhou, 450052 Henan People’s Republic of China; 3 Department of Thyroid Surgery, The First Affiliated Hospital of Zhengzhou University, Zhengzhou, 450052 Henan People’s Republic of China; 4 Interventional Institute of Zhengzhou University, Zhengzhou, 450052 Henan People’s Republic of China; 5 Interventional Treatment and Clinical Research Center of Henan Province, Zhengzhou, 450052 Henan People’s Republic of China; 6 Department of Respiratory and Critical Care Medicine, The First Affiliated Hospital of Zhengzhou University, Zhengzhou, 450052 Henan People’s Republic of China; 7 Department of Neurosurgery, Xiangya Hospital, Central South University, Changsha, 410008 Hunan People’s Republic of China; 8 Department of Oncology, Zhujiang Hospital, Southern Medical University, Guangzhou, 510280 Guangdong People’s Republic of China

**Keywords:** Head and neck squamous cell carcinoma, PI3K pathway mutation, Immunotherapy, Immune environment, Survival

## Abstract

**Background:**

PI3K pathway is the most frequently mutated pathway in head and neck squamous cell carcinoma (HNSC), which plays a crucial role in tumorigenesis and progression. In the present study, we aimed to investigate the role of PI3K pathway mutation in clinical prognosis prediction and the relationship with immune microenvironment and response rate to immunotherapy.

**Methods:**

We collected 129 samples with immunotherapy information from MSKCC-2019 cohort as well as 501 and 40 samples from TCGA-HNSC and MD-Anderson non-immunotherapy cohorts, respectively. Somatic mutation data was utilized to characterize the mutational status of the PI3K pathway. Subsequently, we further analyzed the differences in prognosis, immunotherapy response, genomic alterations, functional characteristics, and immune microenvironment between the mutation and wild groups.

**Results:**

The Kaplan-Meier survival curves displayed that PI3K pathway mutation predicted observably prolonged overall survival (OS) in the immunotherapy cohort MSKCC-2019 (*p* = 0.012) but did not reach statistical significance in the non-immunotherapy cohorts TCGA-HNSC (*p* = 0.68) and MD-Anderson (*p* = 0.68). After incorporating several clinicopathologic features such as age, gender, and tumor mutation burden (TMB), the results of multivariate Cox regression analysis also demonstrated that the PI3K pathway mutation could indicate better immunotherapy outcomes in HNSC patients with a hazard ratio (HR) of 0.533 (95% CI: 0.313–0.910; *p* = 0.021) in the immunotherapy cohort MSKCC-2019, compared with 0.888 (95% CI: 0.636–1.241; *p* = 0.487) and 1.939 (95% CI: 0.483–7.781; *p* = 0.351) in the non-immunotherapy cohorts TCGA-HNSC and MD-Anderson. In addition, the results of the subclass mapping (SubMap) and the tumor immune dysfunction and exclusion (TIDE) also consistently suggested that patients in the mutation group are more likely to benefit from immunotherapy. And further studies showed that the mutation group owned significantly higher TMB, activated immune-related pathways, richer abundance of immune cells, and higher expression levels of immunomodulators. To improve the prognosis of the wild group, we identified five relatively sensitive potential drugs for the wild group, including “BMS-536924,” “linsitinib,” “NVP-TAE684,” “PLX-4720,” and “clonazepam.”

**Conclusions:**

The PI3K pathway mutation status could be considered as a potential biomarker to predict better immunotherapeutic efficacy and clinical outcomes after immunotherapy in HNSC patients.

**Supplementary Information:**

The online version contains supplementary material available at 10.1186/s12957-023-02938-6.

## Background

Head and neck squamous cell carcinoma (HNSC) is the sixth most common cancer worldwide and is mainly induced by smoking, alcohol consumption, and human papillomavirus [1]. Currently, clinical treatment modalities for HNSC include surgical resection, targeted therapy, adjuvant chemotherapy, and radiotherapy [2]. However, due to serious side effects, patients have a limited response rate to these therapeutic measures [3]. In the USA, the 5-year survival rate of HNSC patients is still relatively low, ranging from 40 to 50% [4]. To further improve patient outcomes, more precise patient stratified management and individualized treatment approaches are urgently needed.

The PI3K/AKT pathway is one of the most important pathways regulating many cellular processes, and its abnormal activation leads to cell cycle dysregulation, genomic instability, cell differentiation defects, and persistent mitotic signaling in HNSC patients, thereby enhancing the malignancy of tumors [5]. In terms of the tumor immune microenvironment (TIME), it has been demonstrated that PI3K pathway also regulates many important aspects of immune cell differentiation, development, and function as well as activates numerous immunosuppressive factors, including the expression of immune checkpoints and the infiltration of immunosuppressive cells [6, 7]. In addition, recent studies have illustrated that disrupting the HER3-PI3K-mTOR oncogenic signaling axis reverses the suppressive TIME, enhancing the immunotherapeutic efficacy of PD-1 inhibitors [8–11]. However, the mutation landscape of the PI3K pathway genes in HNSC and the impact of PI3K pathway mutation on the TIME as well as immunotherapy of HNSC remain unknown.

Following the success of immunotherapy in melanoma, an increasing number of clinical trials have confirmed its remarkable efficacy in specific patients [12]. Recently, two phase III randomized trials have revealed that PD-1 inhibitors can prolong patient survival and have better efficacy than chemotherapy in second-line platinum-refractory recurrent/metastatic HNSC [13]. As a classical anti-PD-1 monoclonal antibody, pembrolizumab is superior to cetuximab in combination with chemotherapy in phase III clinical trials and has been approved by the FDA for first-line treatment of recurrent or metastatic HNSC [14]. Several biomarkers, including PD-1, PD-L1, tumor mutation burden (TMB), IFN-γ signature, and HPV factors, have been identified to evaluate the efficacy of immunotherapy in HNSC patients, but only PD-L1 is widely used in clinical practice, and the overall predictive effect is unsatisfactory [15]. Given the relatively high cost and immune-related severe adverse events of immunotherapy, there is an urgent need to explore more accurate and practical predictive markers.

In this study, we characterized PI3K pathway mutation landscape in HNSC patients and analyze its prognostic value in immunotherapy and non-immunotherapy cohorts. After stratifying HNSC patients into the mutation and wild groups, we systematically evaluated the relationship between PI3K pathway mutation and immunological characteristics from multiple dimensions including TMB, immune cell infiltration, immunomodulator expression, and immunotherapy response prediction, respectively. In addition, we also explored potentially sensitive drugs in the wild group population that responded poorly to immunotherapy to significantly improve their prognosis. Overall, our study may provide a reference for early clinical identification of immunotherapy-sensitive HNSC patients to receive further individualized therapy.

## Methods

### Data acquisition and processing

Somatic mutation data and corresponding clinical information for the three independent HNSC cohorts, including the immunotherapy cohort MSKCC-2019 as well as the non-immunotherapy cohorts MD-Anderson and TCGA-HNSC, were obtained from the cBioPortal (https://www.cbioportal.org/) and TCGA (http://portal.gdc.cancer.gov/) databases [16–18]. FPKM-normalized RNA-Seq data of the TCGA-HNSC cohort were acquired from the UCSC-Xena database (http://xena.ucsc.edu/) and further transformed to log2 (TPM + 1).

### Delineation of the PI3K pathway mutation landscape

We obtained 29 PI3K pathway genes from a previous study (Table S1) [19]. The *maftools* package was utilized to extract the mutation status of PI3K pathway genes in the three cohorts, and the mutation landscape of different mutation types was further visualized using the *ComplexHeatmap* package [20, 21].

### Evaluation of prognostic differences between the mutation and wild groups

The 129 samples from MSKCC-2019 cohort, 501 samples from TCGA-HNSC cohort, and 40 samples from MD-Anderson cohort were included in the survival analysis, respectively. Referring to previous criteria, we defined samples with at least one PI3K pathway gene mutated as the PI3K pathway mutation group, otherwise wild group [22, 23]. Multivariate Cox regression and Kaplan-Meier survival analysis were further performed to evaluate prognostic differences between the PI3K pathway mutation and wild groups in the immunotherapy cohort and the non-immunotherapy cohorts.

### Calculation of TMB and acquisition of immune-related indicators

TMB is defined as the total number of base substitutions, insertions, and deletions per million bases in the coding region [24]. The TMB of each sample was calculated by the “tmb” function in the *maftools* package [21]. In addition, we recruited multiple measures, including neoantigens, SNV neoantigens, nonsilent mutation rate, and TCR Shannon from Thorsson V et al., to assess differences in overall genomic instability and immune status between the two groups [25].

### Depicting the genomic alteration landscape

Based on the mutation and copy number alteration (CNA) data from the TCGA-HNSC cohort, we comprehensively analyzed the PI3K pathway mutation and wild groups. The analysis steps are as follows: (1) we first calculated the TMB for each tumor sample and selected the top 15 mutated genes for visualization; (2) referring to the previous study, we extracted the four most common mutational signatures (mutational signatures 1, 2, 4, and 7) in HNSC using the *deconstructSigs* package and further calculated the proportion of their frequencies [26, 27]; (3) after obtaining CNA data from the Firebrowse website (http://firebrowse.org/), we chose chromosome fragments with broad-level CNA frequencies greater than 33% and six classical genes located on chromosomes 7p11.2, 3p24.1, and 9p21.3 for display. In addition, we calculated copy number gain and loss loads occurring at focal and chromosomal arm levels to evaluate global CNA loads between the PI3K pathway mutation and wild groups.

### Functional enrichment analysis

We performed gene set variation analysis (GSVA) and gene set enrichment analysis (GSEA) to explore potential Gene Ontology (GO), Kyoto Encyclopedia of Genes and Genomes (KEGG), and Hallmark pathways associated with PI3K pathway mutation and wild phenotypes in HNSC. Afterward, we selected 10 GSVA pathways to visualize via heatmaps and 5 GSEA pathways to visualize via GSEA plots for the mutation and wild groups, respectively.

### Immune cells infiltration and immunomodulators expression

We applied CIBERSORT and xCell algorithms to evaluate the abundance of immune cell infiltration in the PI3K pathway mutation and wild groups, respectively [28, 29]. In addition, based on Thorsson V et al., we obtained 69 immunomodulators, including 20 co-stimulatory molecules, 12 co-inhibitory molecules, 18 ligand molecules, and 19 receptor molecules [25]. The expression differences of these immunomodulators were further compared between the PI3K pathway mutation and wild groups.

### Assessment of the efficacy of immunotherapy

Using the subclass mapping (SubMap) algorithm [30], an unsupervised method, we compared the similarity of mRNA expression patterns between the PI3K pathway mutation and wild groups in the TCGA-HNSC cohort and immunotherapy-responsive and non-responsive populations from seven immunotherapy cohorts (GSE35640, GSE100797, GSE135222, GSE145996, Nathanson, IMvigor210, and TCGA-SKCM). The tumor immune dysfunction and exclusion (TIDE) algorithm can predict immune checkpoint inhibitors (ICIs) efficacy by modeling the two main mechanisms of tumor immune escape (induction of T cell dysfunction and prevention of T cell infiltration) [31]. We further inferred the response rates to ICIs in the PI3K pathway mutation and wild groups via the TIDE web tool (http://tide.dfci.harvard.edu/).

### Predicting potential therapeutic drugs for the wild group

We then identified potential drugs for the PI3K pathway wild group using the following three steps: (1) the expression data of cancer cell lines (CCLs) were obtained from the Cancer Cell Line Encyclopedia (CCLE) database. The drug-sensitive data with AUC values were obtained from the Cancer Therapeutics Response Portal (CTRP) and PRISM repurposing databases; (2) afterward, we further estimated the drug sensitivity of each sample in the TCGA-HNSC cohort according to the *pRRophetic* package [32]; (3) finally, based on differential drug response analysis, we identified potential drugs sensitive to patients in the PI3K pathway wild group using the following thresholds: log2FC > 0 and *P* < 0.01 for CTRP and PRISM data.

### Statistical analysis

All data processing and analysis for this study were performed using the R 4.1.1 software. Continuous variables between the PI3K pathway mutation and wild groups were analyzed by Student’s *t*-test or Wilcoxon rank-sum test; categorical variables were analyzed by chi-square test or Fisher’s exact test. Differences in Kaplan-Meier survival curves between the two groups were evaluated using the log-rank test. All *P* values were two-sided, and *P* < 0.05 was considered statistically significant.

## Results

### Landscape and prognostic value of PI3K pathway mutation in HNSC

**Fig. 1:**
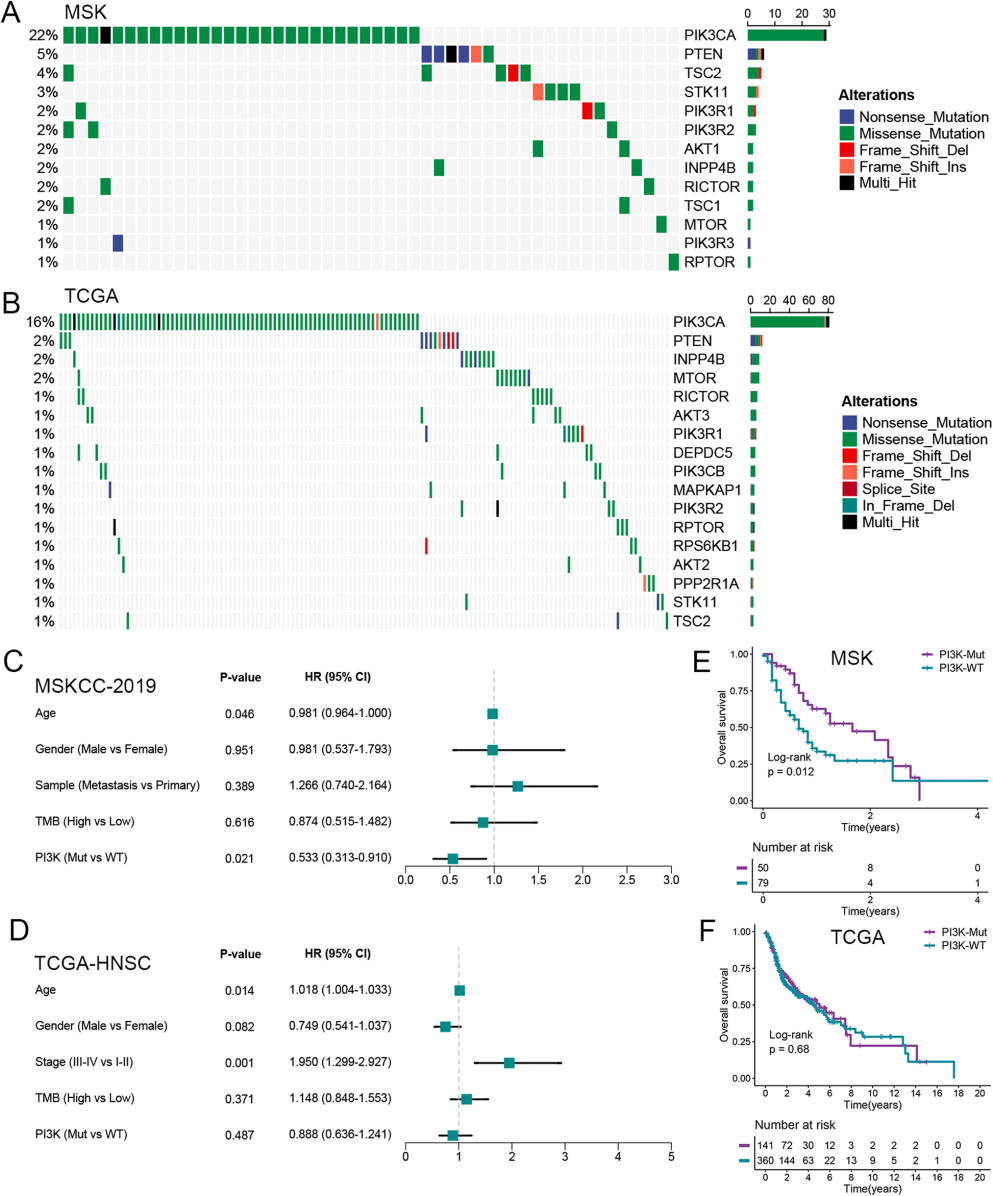
Landscapes and clinical prognostic value of PI3K pathway mutation in HNSC. **A**, **B** Oncoplot depicts the mutated PI3K pathway genes of HNSC in the MSKCC-2019 (**A**) and TCGA-HNSC (**B**) cohorts. The left panel shows the mutation rate, and PI3K pathway genes are ordered by their mutation frequencies. The right panel presents different mutation types. **C**, **D** Multivariate Cox regression analysis of PI3K pathway mutation phenotype. **E**, **F** Kaplan-Meier survival analysis of the PI3K pathway mutation and wild groups in the MSKCC-2019 (*n* = 129) and TCGA-HNSC (*n* = 501) cohorts. WT, wild type; Mut, mutant type

The workflow for this study is shown in Fig. S1. A total of 13, 17, and 3 PI3K pathway mutated genes were identified in the MSKCC-2019, TCGA-HNSC, and MD-Anderson cohorts (Figs. 1A, B and S2A). Multivariate Cox regression analysis showed that the hazard ratio (HR) of PI3K pathway mutation phenotype in the immunotherapy cohort MSKCC-2019 was 0.533 (95% CI: 0.313–0.910; *p* = 0.021), whereas the HRs in the non-immunotherapy cohorts TCGA-HNSC and MD-Anderson were 0.888 (95% CI: 0.636–1.241; *p* = 0.487) and 1.939 (95% CI: 0.483–7.781; *p* = 0.351), respectively (Figs. 1C, D and S2B). In addition, Kaplan-Meier survival analysis was further performed to determine whether the PI3K pathway mutation phenotype was associated with OS and DFS (disease-free survival). Similarly, patients with PI3K pathway mutation had significantly prolonged OS in the immunotherapy cohort MSKCC-2019 (*n* = 129), but there were not statistically significantly associated with OS and DFS in the non-immunotherapy cohorts TCGA-HNSC (*n* = 501) and MD-Anderson (*n* = 40; Figs. 1E, F and S2C-D).

### Genomic alteration landscape between the mutation and wild groups

**Fig. 2:**
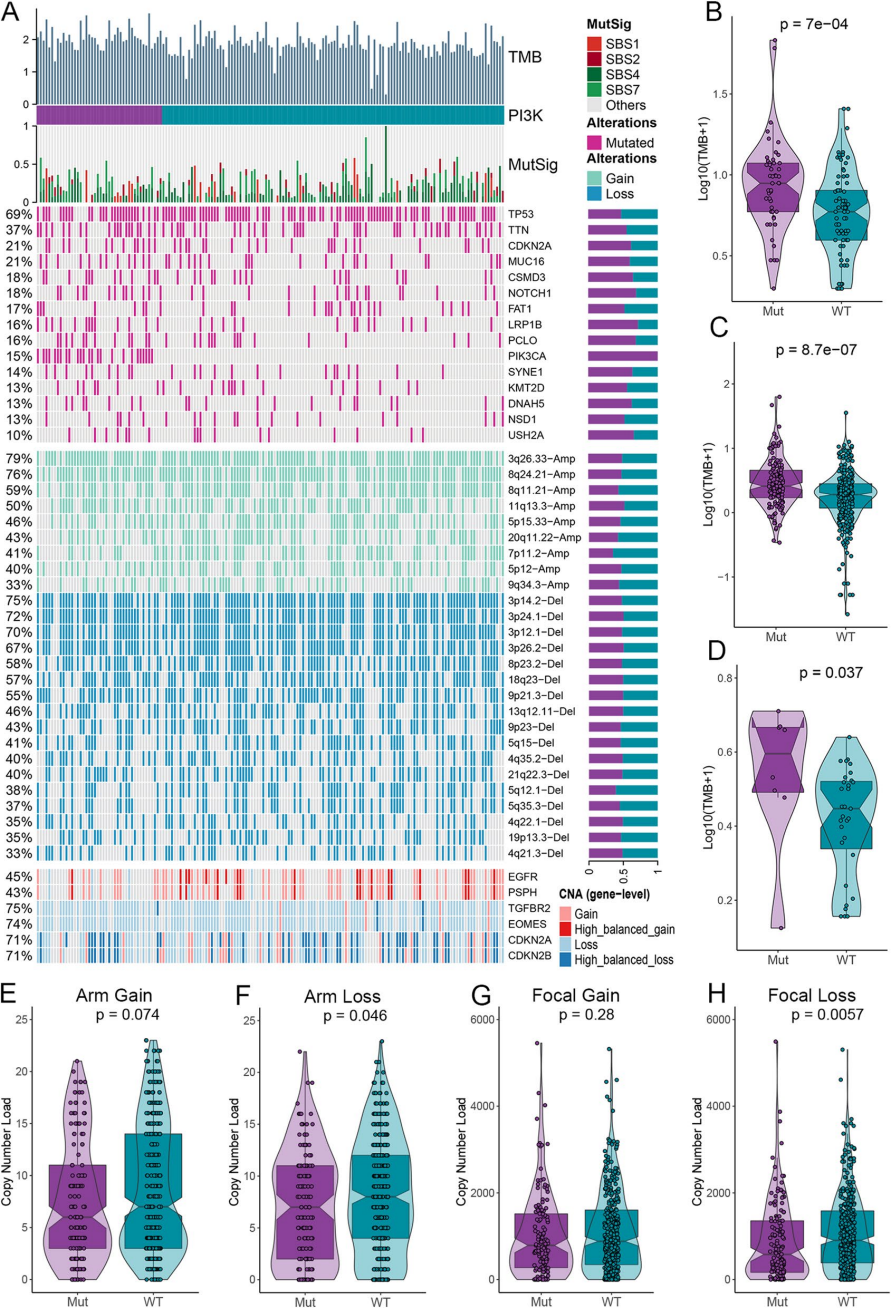
Genomic alteration landscape. **A** Genomic alteration landscape of the PI3K pathway mutation and wild groups. TMB, patient subgroups, the relative contribution of four mutational signatures, selected top 15 mutated genes, broad-level CNAs (> 33%), and selected genes located within chromosomes 7p11.2, 3p24.1, and 9p21.3 are shown from the top to the bottom panels. The relative proportions for the frequencies of each alteration between the PI3K pathway mutation and wild groups are presented in the right bar charts. **B**–**D** The PI3K pathway mutation group is significantly associated with higher TMB in the MSKCC-2019 (**B**), TCGA-HNSC (**C**), and MD-Anderson (**D**) cohorts. **E**–**H** Differences in copy number amplification loads at chromosomal arm (**E**) and focal (**G**) levels, as well as differences in copy number deletion loads at chromosomal arm (**F**) and focal (**H**) levels between the two groups in the TCGA-HNSC cohort. WT, wild type; Mut, mutant type

After stratifying HNSC patients into the PI3K pathway mutation and wild groups, the results of all three cohorts exhibited significantly higher TMB in the PI3K pathway mutation group than in the wild group (Fig. 2B–D). In addition, we chose the top 15 mutated genes of the TCGA-HNSC cohort further to explore the mutation landscape of the two groups. The results showed that the mutations in tumor suppressor gene CDKN2A and oncogenes TTN, MUC16, and NOTCH1 were more common in the mutation group (Fig. 2A). In addition, CNA analysis at focal and chromosomal arm levels exhibited a higher frequency of copy number deletions in the wild group (*P* < 0.05), while copy number amplifications failed to reach statistical significance, although there was a corresponding trend (*P* > 0.05; Fig. 2A and E–H). These results were also confirmed at the gene level by high-frequency amplifications of oncogenes (EGFR and PSPH) and high-frequency deletions of tumor suppressor genes (TGFBR2, EOMES, CDKN2A, and CDKN2B) in the PI3K pathway wild group (Fig. 2A).

### Potential biological mechanisms of the PI3K pathway mutation and wild groups

**Fig. 3:**
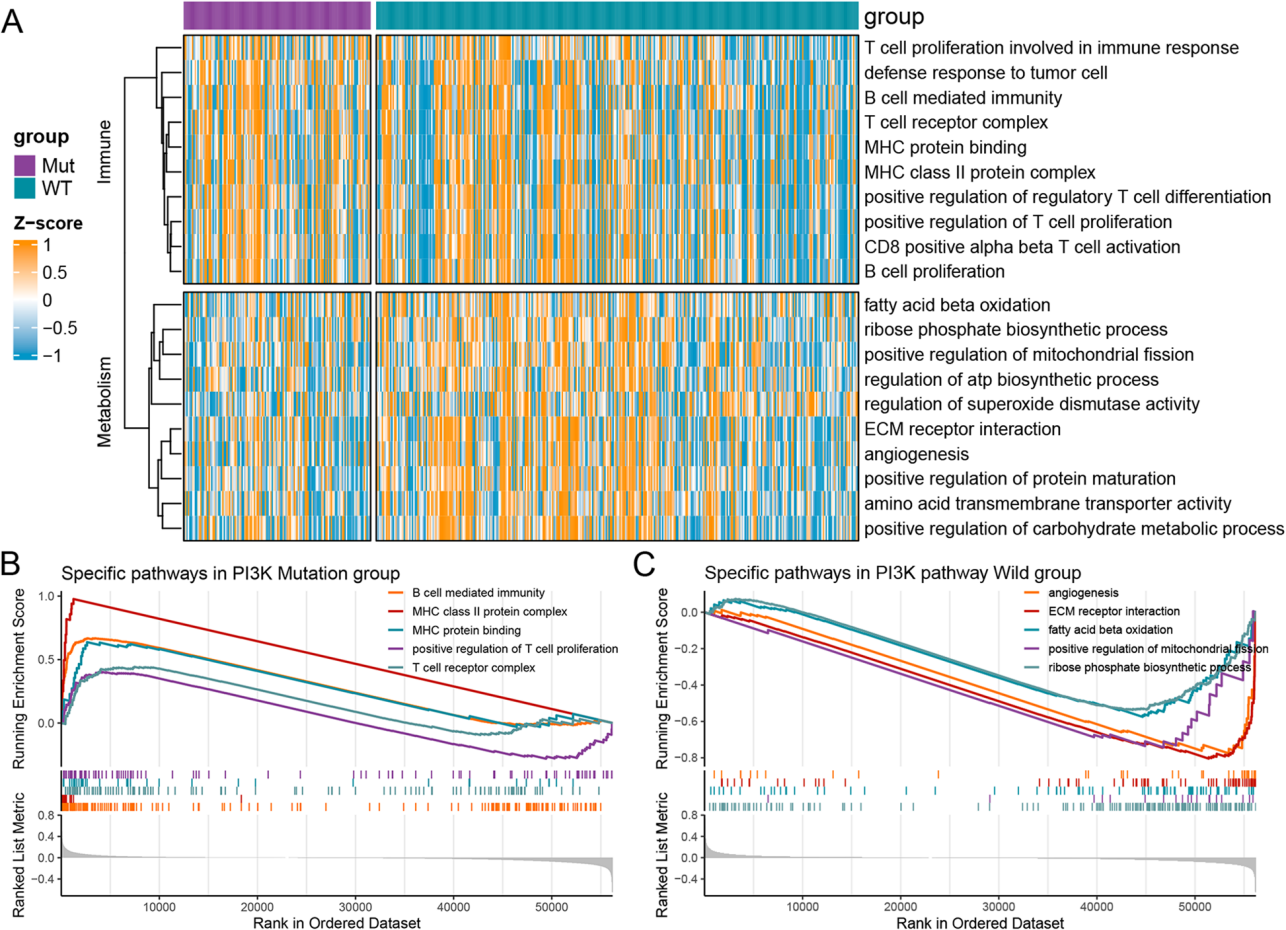
Functional enrichment analysis. **A** Ten GO, KEGG, and Hallmark pathways enriched by GSVA analysis in the PI3K pathway mutation and wild groups, respectively. **B**, **C** GSEA analysis showed the top five pathways enriched in the PI3K pathway mutation group (**B**) and the top five pathways enriched in the wild group (**C**). GSVA, gene set variation analysis; GSEA, gene set enrichment analysis

GSVA enrichment analysis integrating GO, KEGG, and Hallmark pathways indicated that immune-related pathways were significantly enriched in the PI3K pathway mutation group, while the PI3K pathway wild group was closely related to metabolism-related pathways (Fig. 3A). Furthermore, as shown in Fig. 3B, the GSEA analysis result also demonstrated that the PI3K pathway mutation group was prominently enriched for immune pathways, such as B cell-mediated immunity (NES = 2.334, FDR < 0.01), MHC class II protein complex (NES = 2.200, FDR < 0.01), MHC protein binding (NES = 1.791, FDR < 0.01), T cell receptor complex (NES = 1.503, FDR < 0.01), and positive regulation of T cell proliferation (NES = 1.329, FDR < 0.01), while the PI3K pathway wild group was significantly correlated with extracellular matrix (ECM) receptor interaction (NES = − 2.064, FDR < 0.01), angiogenesis (NES = − 1.773, FDR < 0.01), positive regulation of mitochondrial fission (NES = − 1.509, FDR < 0.01), ribose phosphate biosynthetic process (NES = − 1.437, FDR < 0.01), and fatty acid beta oxidation (NES = − 1.428, FDR < 0.01; Fig. 3C).

### Immune cells infiltration and immunomodulators expression

**Fig. 4:**
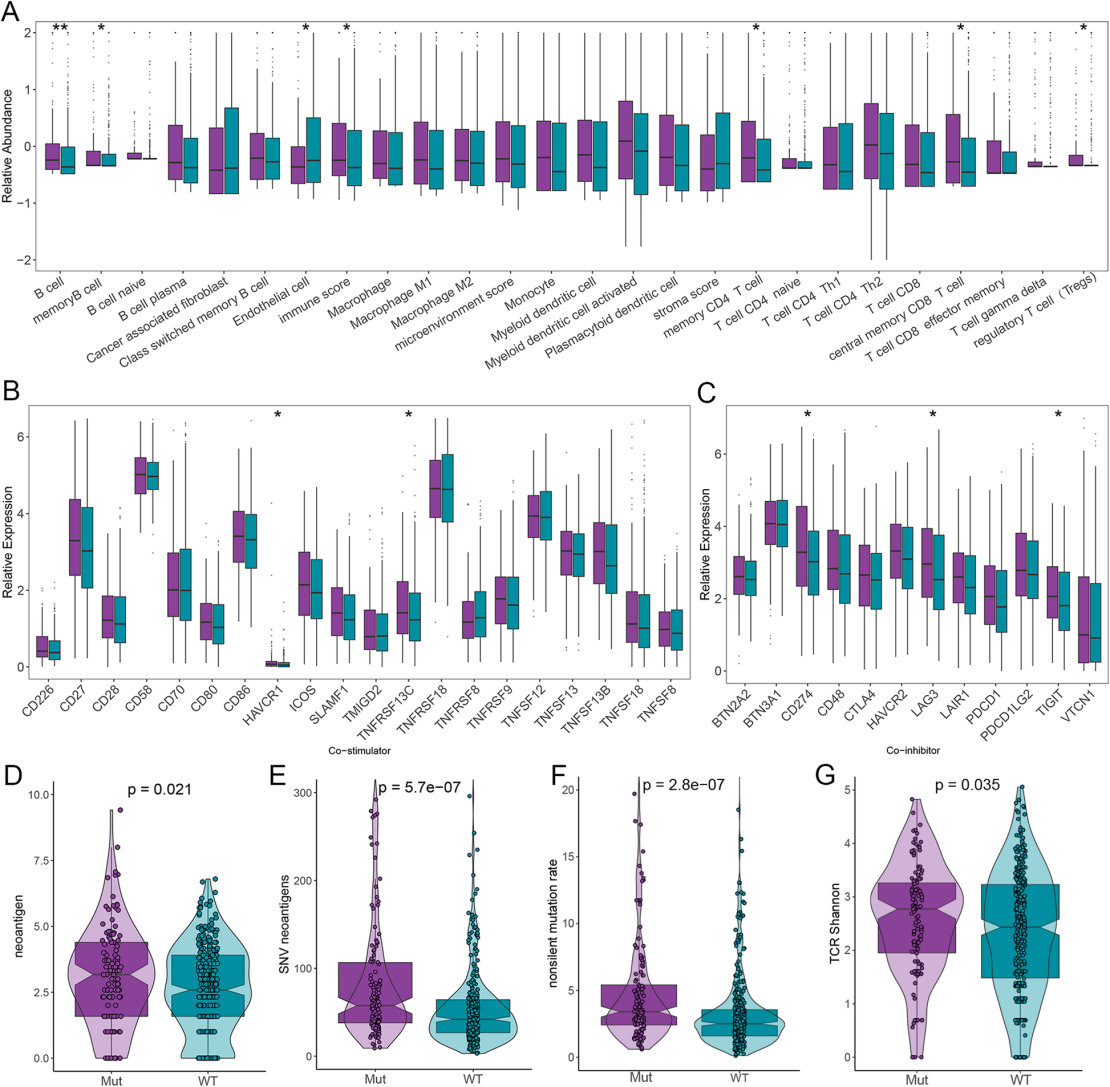
The immune landscape of the PI3K pathway mutation and wild groups in the TCGA-HNSC cohort. **A** Distribution of 26 immune and stromal cell infiltration calculated by xCell in the PI3K pathway mutation and wild groups. **B**, **C** Boxplots represent different expression levels of 20 co-stimulatory molecules (**B**) and 12 co-inhibitory molecules (**C**) between the two groups. **D**–**G** Boxplots of neoantigen (**D**), SNV neoantigens (**E**), nonsilent mutation rate (**F**), and TCR Shannon (**G**) for patients in the two groups. WT, wild type; Mut, mutant type. *P < 0.05; **P < 0.01

The results of xCell and CIBERSORT consistently demonstrated that the PI3K pathway mutation group possessed superior immune effector and immunosuppressive cells infiltration, including B cell, memory B cell, memory CD4 T cell, central memory CD8 T cell, and regulatory T cell (Treg). However, stromal components were more enriched in the PI3K pathway wild group (such as endothelial cell; Figs. 4A and S2E-F). In addition to abundant immune cells, the mutation group also exhibited higher levels of co-stimulatory molecules (such as HAVCR1 and TNFRSF13C) and co-inhibitory molecules (such as CD274, LAG3, TIGIT) expression (Fig. 4B, C). We have also observed corresponding trends in other molecules that play a key role in anti-tumor immunity and immunotherapy, such as ligand molecules and receptor molecules (Figs. S2G and S3A). Furthermore, several critical indicators for evaluating tumor immunogenicity and antigen presentation efficiency collected from the study of Thorsson et al. also indicated that the PI3K pathway mutation group displayed significantly higher neoantigen, SNV neoantigens, nonsilent mutation rate, and TCR Shannon (Fig. 4D–G) [25].

### Assessment of immunotherapy as well as chemotherapy

**Fig. 5:**
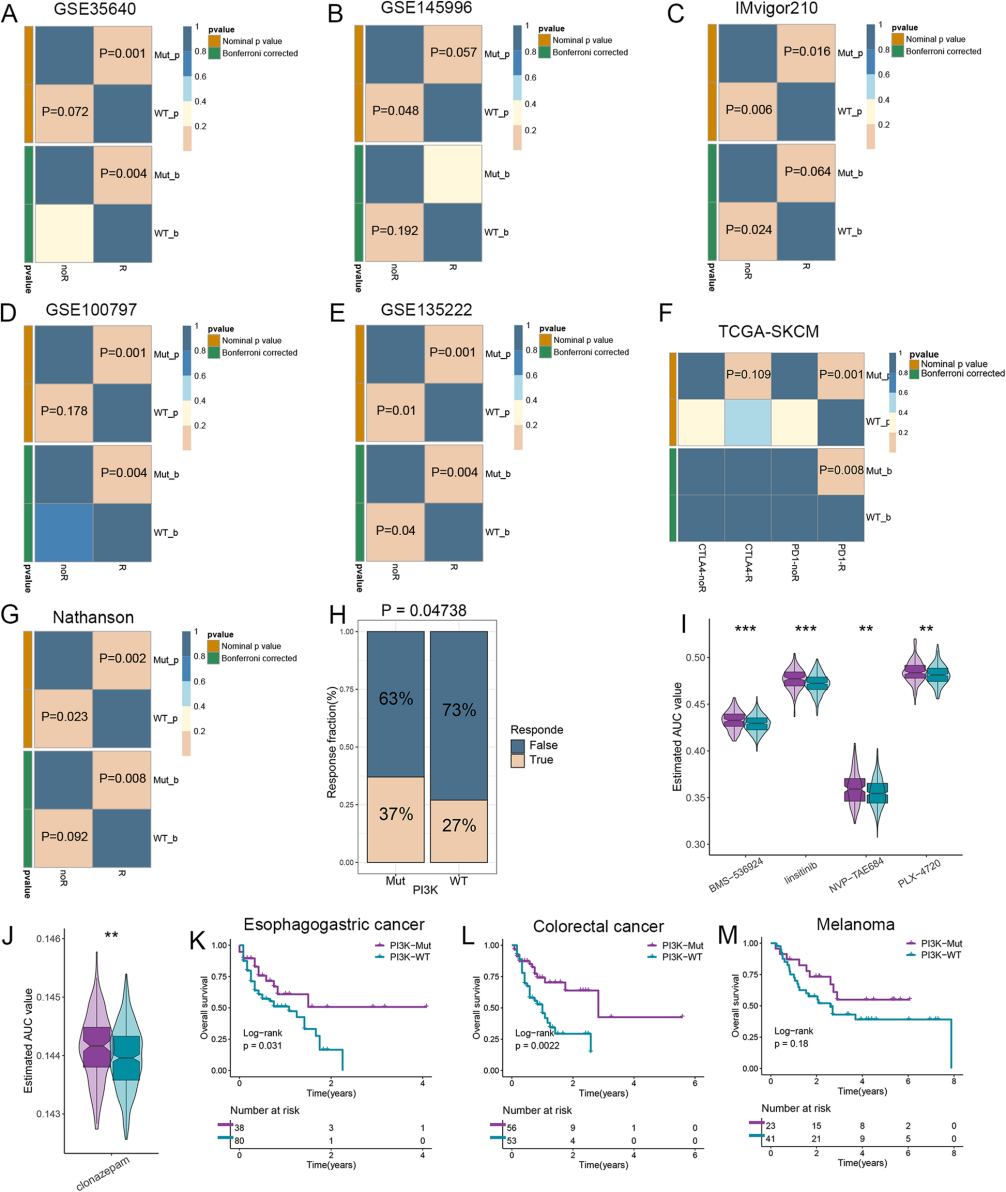
Assessment of immunotherapy and chemotherapy in the PI3K pathway mutation and wild groups as well as pan-cancer prognostic landscape. **A**–**G** The SubMap algorithm evaluated the expression similarity between patients in the PI3K pathway mutation and wild groups and patients with different immunotherapy responses. **H** The TIDE algorithm predicted the distribution of immunotherapy responders in the two groups. **I**, **J** Five potential antitumor drugs specifically sensitive to patients in the PI3K pathway wild group were identified using drug sensitivity data from the CTRP (**I**) and PRISM (**J**) databases. **K**–**M** Kaplan-Meier survival analysis of the PI3K pathway mutation and wild groups in esophagogastric cancer (**K**), colorectal cancer (**L**), and melanoma (**M**). SubMap, subclass mapping; TIDE, tumor immune dysfunction and exclusion. *P < 0.05; **P < 0.01; ***P < 0.001

The SubMap analysis demonstrated that the PI3K pathway mutation group was genetically more similar to patients responding to immunotherapy, particularly anti-PD-L1 therapy (*P* < 0.05; Fig. 5A–G). In parallel, TIDE also revealed a significantly higher response rate to immunotherapy in the PI3K pathway mutation group (*P* < 0.05; Fig. 5H). In addition, using drug-sensitive data from the CTRP and PRISM databases combined with CCLs expression data from the CCLE database, we further developed five potential therapeutic drugs for the PI3K pathway wild group, including “BMS-536924,” “linsitinib,” “NVP-TAE684,” “PLX-4720,” and “clonazepam” (*P* < 0.01; Fig. 5I–J).

### Prognostic performance in pan-cancer

Given the accurate predictive power of the PI3K pathway mutation for immunotherapy in HNSC, we further explored its significance in other common tumors based on pan-cancer genomic data and immunotherapy information from the MSKCC-2019 cohort (esophagogastric cancer and colorectal cancer) and MSKCC-2014 cohort (melanoma). As shown in Fig. 5K–L, the results of Kaplan-Meier survival analysis revealed a significantly prolonged OS in immunotherapy cohorts of esophagogastric cancer (*P* = 0.031) and colorectal cancer (*P* = 0.0022). It is worth pointing out that since the MSKCC-2014 cohort had only 64 melanoma samples, although their Kaplan-Meier curves prominently separated, the result did not reach statistical significance (*P* = 0.18; Fig. 5M).

### Correlation between PI3K pathway mutation with HPV status and patient prognosis

Given that numerous studies have demonstrated that human papillomavirus (HPV) infection status is a favorable prognostic factor in HNSC patients [33, 34], based on HPV status information from the TCGA-HNSC cohort, we further explored the relationship between the PI3K pathway mutation and HPV status and patient prognosis. As exhibited in Fig. S3B-C, we calculated the proportion of HPV status in the PI3K pathway wild and mutation group and the proportion of PI3K pathway mutation in patients with different HPV status, and the differences did not reach statistical significance. On the other hand, the PI3K pathway mutation status was unable to observably stratify OS in HNSC patients (*P* = 0.68); however, HPV-positive status was associated with significantly prolonged OS (*P* = 0.04; Fig. 1F and S3D). In addition, we performed Kaplan-Meier survival analysis of PI3K pathway mutation in HPV-negative patients (among the 19 HPV-positive HNSC patients, only 1 of 12 PI3K wild-type patients died, while 7 PI3K pathway mutated patients did not even die, and further subgroup analysis could not be performed). The results demonstrated that when we stratified patients according to HPV status, there was no significant difference in the effect of PI3K pathway mutation on OS, and the differences did not reach statistical significance (*P* = 0.62; Fig. S3E). Finally, we performed Kaplan-Meier survival analysis of HPV status in patients with PI3K pathway mutation and wild group separately (Fig. S3F-G). Consistent with Fig. S3D, HPV-positive patients owned prolonged OS in both PI3K pathway mutation and wild groups, but the differences did not reach statistical significance limited to their small sample size. Overall, these results certified that HPV status can predict a better prognosis in HNSC patients and is not affected by PI3K pathway mutation status.

## Discussion

HNSC is the sixth most common cancer worldwide. Currently, treatment modalities for HNSC include surgical resection, targeted therapy, adjuvant chemotherapy, radiotherapy, and immunotherapy [35, 36]. Mutation status of particular genes could predict therapy outcomes, for example, mutation of the p53 gene is associated with an increased risk of locoregional failure in patients with invasive HNSC who are treated with radiation therapy, PIK3CA mutation predicted worse DFS in a prospective cohort of HPV-associated oropharyngeal squamous cell carcinoma patients treated with definitive chemoradiation, mutation in the epidermal growth factor receptor ligand-binding domain confers increased sensitivity to cetuximab, and TMB and PRKDC mutation are predictive biomarkers for immunotherapy [18, 37–41]. However, the predictive ability of these biomarkers to ICIs response is still limited. We then explored the relationship between PI3K pathway mutation, the most frequently mutated pathway in HNSC, and the immune microenvironment as well as immunotherapeutic efficacy.

In this study, we divided HNSC patients into the mutation and wild groups according to the mutation status of PI3K pathway genes and found that the mutation group had longer OS observably in the immunotherapy cohort. However, the mutation status was not significantly associated with OS and DFS in the non-immunotherapy cohorts. Further studies exhibited that the PI3K pathway mutation group owned significantly higher TMB as well as mutation frequencies of oncogenes and tumor suppressor genes, relatively active immune-related pathways, richer immune cell infiltration, and higher expression levels of immunomodulators. The predicted results of TIDE and SubMap consistently indicated that HNSC patients in the PI3K pathway mutation group were more likely to benefit from immunotherapy. Thus, PI3K pathway mutation status may be a reliable biomarker to predict the efficacy of immunotherapy in HNSC patients.

In HNSC patients, aberrant activation of the PI3K/AKT/MTOR pathway promotes malignant progression and impacts many vital aspects of immune cell development, differentiation, and activation of immunosuppressive factors [5–7, 42]. Moreover, previous studies have demonstrated that the PI3K pathway is the most common mutant oncogenic pathway (30.5%) and predicts a higher mutation rate in HNSC [43]. We, therefore, investigated the landscape and prognostic value of PI3K pathway mutation in HNSC. The results of all three cohorts consistently indicated that PI3K pathway genes are commonly mutated (38.8%, 28.1%, and 20%; Figs. 1A, B and S2A). Kaplan-Meier curves and multivariate Cox regression analyses demonstrated that patients who harbored PI3K pathway mutation had significantly prolonged OS in the immunotherapy cohort. Interestingly, there were no statistical differences in OS and DFS between the PI3K pathway mutation and wild groups in the non-immunotherapy cohorts. This suggested that PI3K pathway mutation may herald a better efficacy of immunotherapy in HNSC patients, and it may serve as a potential biomarker to select immunotherapy-sensitive patients for further immunotherapy.

To explore the potential mechanisms that may contribute to the differential efficacy of immunotherapy and prognosis in patients with PI3K pathway mutation and wild groups, we performed an integrated genomic variation analysis between the two groups. Consistent with Lui et al., the mutation group possessed significantly higher mutation rates of tumor suppressor gene CDKN2A and oncogenes TTN, NOTCH1, and MUC16 [43]. Many studies have confirmed that higher TMB in solid tumors predicts better immunotherapeutic outcomes, and the significantly higher TMB in the PI3K pathway mutation group of the three cohorts also supports its better immunotherapeutic efficacy and prognosis in the immunotherapy cohort [44–46]. In contrast, CNA analysis showed increased copy number amplification and deletion loads at both focal and chromosomal arm levels in the PI3K pathway wild group, suggesting that CNA may play a key role in malignant progression in the wild group. The two malignant biological behaviors, gene mutation, and CNA, predominated in the PI3K pathway mutation and wild groups, respectively, which is consistent with the fact that there were no differences in prognosis between the two groups in the non-immunotherapy cohorts TCGA-HNSC and MD-Anderson [47–49].

Next, we performed GSVA and GSEA enrichment analyses separately to explore the underlying biological mechanisms of the PI3K pathway mutation and wild groups. The results exhibited that the immune-related pathways, including B cell-mediated immunity, MHC protein binding, T cell receptor complex, and positive regulation of T cell proliferation, were significantly enriched in the PI3K pathway mutation group. On the other hand, specific pathways associated with malignant tumor progression such as angiogenesis, positive regulation of mitochondrial fission, and metabolism-related pathways such as fatty acid beta oxidation were relatively activated in the PI3K pathway wild group, indicating that we can develop specific drugs targeting tumor angiogenesis, cell proliferation, and metabolism for patients in the wild group.

Considering the significant enrichment of immune-related biological processes in the PI3K pathway mutation group, we further evaluated the abundance of immune cell infiltration and immunomodulators expression levels between the two groups. The results from xCell and CIBERSORT consistently indicated that killer cells such as memory CD4 T cells and central memory CD8 T cells, which play critical roles in anti-tumor immunity and immunotherapy, were significantly richer in the PI3K pathway mutation group. Similarly, the expression levels of immunomodulators, including co-stimulatory, co-inhibitory, ligand, and receptor molecules, were also significantly higher in the PI3K pathway mutation group. Thus, we have reason to believe that anti-tumor immunity will be more active in the PI3K pathway mutation group after targeting these immunomodulators using ICIs, and reactivated killer immune cells will exert anti-tumor function and improve patient outcomes [50–52].

Our previous findings exhibited that the PI3K pathway mutation group possessed significantly higher levels of TMB, killer immune cell infiltration, and immunomodulators expression, hinting that patients in the mutation group are more sensitive to immunotherapy [18, 45, 50, 53, 54]. The results from TIDE and SubMap algorithms also consistently demonstrated that patients in the PI3K pathway mutation group were more likely to benefit from immunotherapy. On the other hand, using drug sensitivity data from CTRP and PRISM databases, we identified five potential therapeutic drugs for HNSC patients in the PI3K pathway wild group who did not respond well to immunotherapy. Among them, “BMS-536924” and “linsitinib” are IGF-1R/IR inhibitors that can inhibit glucose metabolism in tumor cells and then exert anti-tumor effects [55, 56]. This is consistent with the significant enrichment of metabolism-related biological pathways in the PI3K pathway wild group and provides a reference for treating patients with relatively poor prognosis in the wild group.

This study is the first to elaborate the mutation landscape of the PI3K pathway in HNSC and found that PI3K pathway mutation predicts better immunotherapeutic efficacy and significantly prolonged OS in HNSC patients after receiving immunotherapy. Second, we explored the utility and generalizability of our findings and confirmed that PI3K pathway mutation could still accurately predict better immunotherapeutic outcomes in other common tumors such as esophagogastric and colorectal cancers. However, our study also has some shortcomings. For instance, due to the lack of immunotherapy cohorts for HNSC, we collected only 129 samples with complete mutation, immunotherapy, and prognosis information from the MSKCC-2019 cohort, and more multi-center prospective studies are needed to validate our results in the future.

## Conclusion

Based on the mutation status of PI3K pathway genes, we found that PI3K pathway mutation predicted a better prognosis for HNSC patients in the immunotherapy cohort, whereas there was no prognostic difference in the non-immunotherapy cohorts. The PI3K pathway mutation group owned significantly higher TMB as well as mutation frequencies of oncogenes and tumor suppressor genes, enriched immune-related pathways, more affluent abundance of immune cells infiltration, and superior immunomodulators expression and was more sensitive to immunotherapy. Correspondingly, the PI3K pathway wild group had a relatively high copy number amplification and deletion loads, mainly enriched in metabolism-related pathways, and thereby we identified five potential specific drugs for this property. Overall, PI3K pathway mutation may be an underlying biomarker for immunotherapy in HNSC patients and could provide a reference for early identifying immunotherapy-sensitive populations to receive further immunotherapy.

## Supplementary Information

**Additional file 1: Fig. S1.** Overall workflow diagram of our research. **Fig. S2.** Landscape and clinical prognostic value of PI3K pathway mutation in the MD-Anderson cohort as well as heatmaps of immune cells infiltration and boxplots of immunomodulators expression. (A) Oncoplot depicts the landscape of PI3K pathway gene mutation in the MD-Anderson cohort. (B) Multivariate Cox regression analysis of PI3K pathway mutation phenotype in the MD-Anderson cohort. (C-D) Kaplan-Meier survival analysis of OS (C) and DFS (D) between the PI3K pathway mutation and wild groups in the MD-Anderson cohort. (E-F) Assess infiltration abundance of 22 immune cells calculated by CIBERSORT (E) as well as 39 immune cells and stromal cells calculated by xCell (F). (G) Boxplots represent different expression levels of 18 ligand molecules between the two groups. WT, wild type; MT, mutant type; *P < 0.05. **Fig. S3.** The relationship between PI3K pathway mutation and HPV status and patient prognosis in the TCGA-HNSC cohort. (A) Boxplots represent different expression levels of 19 receptor molecules between the PI3K pathway mutation and wild groups. (B) Composition percentage of HPV status between the PI3K pathway mutation and wild groups. (C) Composition percentage of PI3K pathway mutation status between the HPV-negative and HPV-positive groups. (D) Kaplan-Meier survival analysis of the HPV status in the TCGA-HNSC cohort. (E) Kaplan-Meier survival analysis of PI3K pathway mutation in the HPV-negative group patients of TCGA-HNSC cohort. (F-G) Kaplan-Meier survival analysis of HPV status in the PI3K pathway wild (F) and mutation (G) group patients of TCGA-HNSC cohort. WT, wild type; MT, mutant type; *P < 0.05; **P < 0.01. **Table S1.** The 29 PI3K pathway genes used to define samples as PI3K pathway mutation or wild groups.

## Data Availability

Public data used in this work can be acquired from the TCGA Research Network portal (https://portal.gdc.cancer.gov/), the cBioPortal (https://www.cbioportal.org/), and the UCSC-Xena database (http://xena.ucsc.edu/). Other data supporting the findings of this study are available from the corresponding author upon reasonable request.

## Abbreviations

HNSC: Head and neck squamous cell carcinoma
TIME: Tumor immune microenvironment
CNA: Copy number alteration
GSVA: Gene set variation analysis
GSEA: Gene set enrichment analysis
GO: Gene Ontology
KEGG: Kyoto Encyclopedia of Genes and Genomes
SubMap: Subclass mapping
TIDE: The tumor immune dysfunction and exclusion
ICIs: Immune checkpoint inhibitors
CCLs: Cancer cell lines
CCLE: Cancer Cell Line Encyclopedia
CTRP: Cancer Therapeutics Response Portal
FDR: False discovery rate
HR: Hazard ratio
DFS: Disease-free survival
HPV: Human papillomavirus
