# Supplementary figures and images for: PI3K pathway mutation predicts an activated immune microenvironment and better immunotherapeutic efficacy in head and neck squamous cell carcinoma
Source: World J Surg Oncol. 2023 Mar 2;21:72. doi: 10.1186/s12957-023-02938-6 (PMC9979448; doi:10.1186/s12957-023-02938-6)

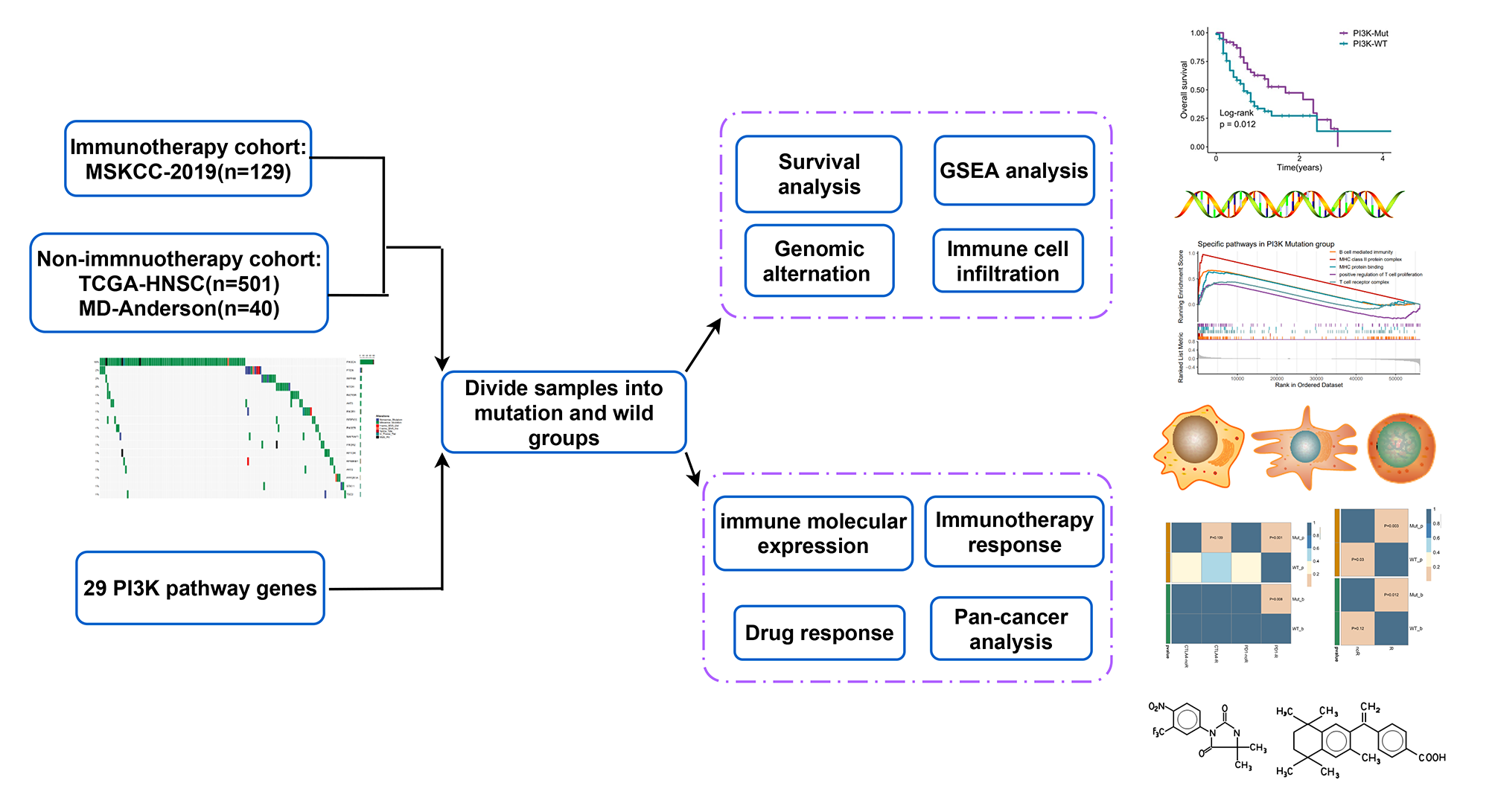

Supplement: Supplementary file 1 — Additional file 1: Fig. S1. Overall workflow diagram of our research. Fig. S2. Landscape and clinical prognostic value of PI3K pathway mutation in the MD-Anderson cohort as well as heatmaps of immune cells infiltration and boxplots of immunomodulators expression. (A) Oncoplot depicts the landscape of PI3K pathway gene mutation in the MD-Anderson cohort. (B) Multivariate Cox regression analysis of PI3K pathway mutation phenotype in the MD-Anderson cohort. (C-D) Kaplan-Meier survival analysis of OS (C) and DFS (D) between the PI3K pathway mutation and wild groups in the MD-Anderson cohort. (E-F) Assess infiltration abundance of 22 immune cells calculated by CIBERSORT (E) as well as 39 immune cells and stromal cells calculated by xCell (F). (G) Boxplots represent different expression levels of 18 ligand molecules between the two groups. WT, wild type; MT, mutant type; *P < 0.05. Fig. S3. The relationship between PI3K pathway mutation and HPV status and patient prognosis in the TCGA-HNSC cohort. (A) Boxplots represent different expression levels of 19 receptor molecules between the PI3K pathway mutation and wild groups. (B) Composition percentage of HPV status between the PI3K pathway mutation and wild groups. (C) Composition percentage of PI3K pathway mutation status between the HPV-negative and HPV-positive groups. (D) Kaplan-Meier survival analysis of the HPV status in the TCGA-HNSC cohort. (E) Kaplan-Meier survival analysis of PI3K pathway mutation in the HPV-negative group patients of TCGA-HNSC cohort. (F-G) Kaplan-Meier survival analysis of HPV status in the PI3K pathway wild (F) and mutation (G) group patients of TCGA-HNSC cohort. WT, wild type; MT, mutant type; *P < 0.05; **P < 0.01. Table S1. The 29 PI3K pathway genes used to define samples as PI3K pathway mutation or wild groups. [file 12957_2023_2938_MOESM1_ESM.zip › Figure S1.tif]

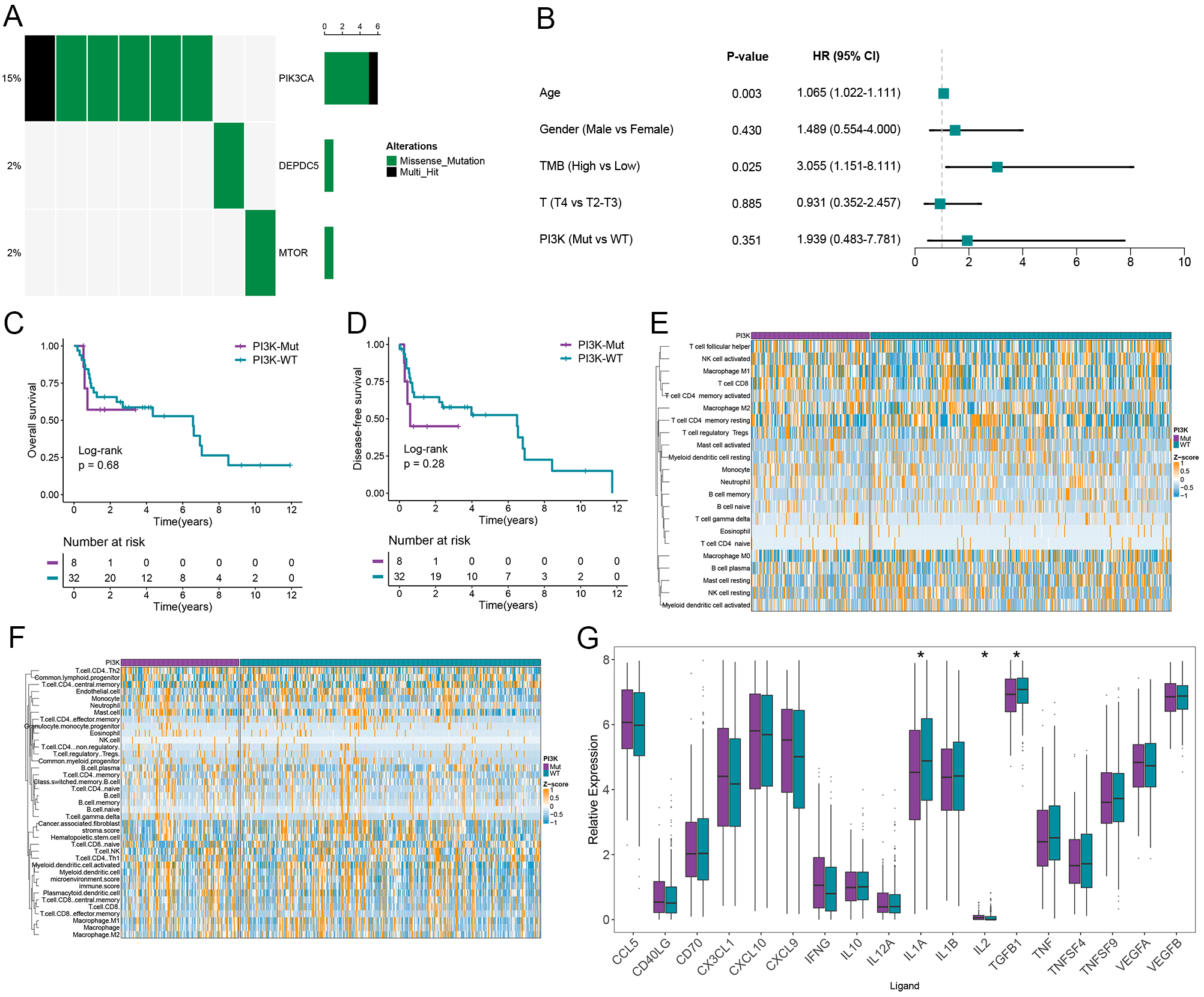

Supplement: Supplementary file 1 — Additional file 1: Fig. S1. Overall workflow diagram of our research. Fig. S2. Landscape and clinical prognostic value of PI3K pathway mutation in the MD-Anderson cohort as well as heatmaps of immune cells infiltration and boxplots of immunomodulators expression. (A) Oncoplot depicts the landscape of PI3K pathway gene mutation in the MD-Anderson cohort. (B) Multivariate Cox regression analysis of PI3K pathway mutation phenotype in the MD-Anderson cohort. (C-D) Kaplan-Meier survival analysis of OS (C) and DFS (D) between the PI3K pathway mutation and wild groups in the MD-Anderson cohort. (E-F) Assess infiltration abundance of 22 immune cells calculated by CIBERSORT (E) as well as 39 immune cells and stromal cells calculated by xCell (F). (G) Boxplots represent different expression levels of 18 ligand molecules between the two groups. WT, wild type; MT, mutant type; *P < 0.05. Fig. S3. The relationship between PI3K pathway mutation and HPV status and patient prognosis in the TCGA-HNSC cohort. (A) Boxplots represent different expression levels of 19 receptor molecules between the PI3K pathway mutation and wild groups. (B) Composition percentage of HPV status between the PI3K pathway mutation and wild groups. (C) Composition percentage of PI3K pathway mutation status between the HPV-negative and HPV-positive groups. (D) Kaplan-Meier survival analysis of the HPV status in the TCGA-HNSC cohort. (E) Kaplan-Meier survival analysis of PI3K pathway mutation in the HPV-negative group patients of TCGA-HNSC cohort. (F-G) Kaplan-Meier survival analysis of HPV status in the PI3K pathway wild (F) and mutation (G) group patients of TCGA-HNSC cohort. WT, wild type; MT, mutant type; *P < 0.05; **P < 0.01. Table S1. The 29 PI3K pathway genes used to define samples as PI3K pathway mutation or wild groups. [file 12957_2023_2938_MOESM1_ESM.zip › Figure S2.tif]

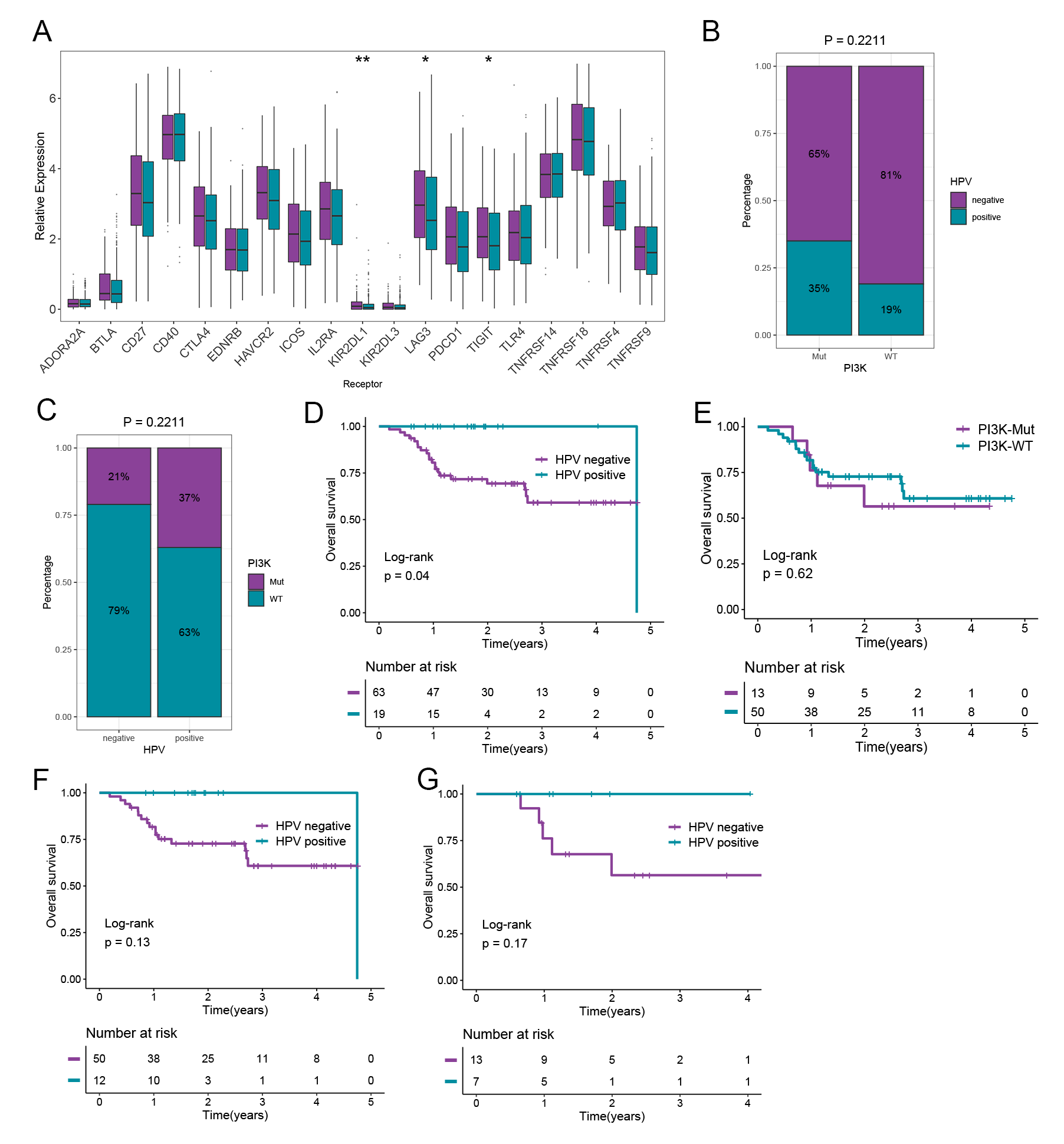

Supplement: Supplementary file 1 — Additional file 1: Fig. S1. Overall workflow diagram of our research. Fig. S2. Landscape and clinical prognostic value of PI3K pathway mutation in the MD-Anderson cohort as well as heatmaps of immune cells infiltration and boxplots of immunomodulators expression. (A) Oncoplot depicts the landscape of PI3K pathway gene mutation in the MD-Anderson cohort. (B) Multivariate Cox regression analysis of PI3K pathway mutation phenotype in the MD-Anderson cohort. (C-D) Kaplan-Meier survival analysis of OS (C) and DFS (D) between the PI3K pathway mutation and wild groups in the MD-Anderson cohort. (E-F) Assess infiltration abundance of 22 immune cells calculated by CIBERSORT (E) as well as 39 immune cells and stromal cells calculated by xCell (F). (G) Boxplots represent different expression levels of 18 ligand molecules between the two groups. WT, wild type; MT, mutant type; *P < 0.05. Fig. S3. The relationship between PI3K pathway mutation and HPV status and patient prognosis in the TCGA-HNSC cohort. (A) Boxplots represent different expression levels of 19 receptor molecules between the PI3K pathway mutation and wild groups. (B) Composition percentage of HPV status between the PI3K pathway mutation and wild groups. (C) Composition percentage of PI3K pathway mutation status between the HPV-negative and HPV-positive groups. (D) Kaplan-Meier survival analysis of the HPV status in the TCGA-HNSC cohort. (E) Kaplan-Meier survival analysis of PI3K pathway mutation in the HPV-negative group patients of TCGA-HNSC cohort. (F-G) Kaplan-Meier survival analysis of HPV status in the PI3K pathway wild (F) and mutation (G) group patients of TCGA-HNSC cohort. WT, wild type; MT, mutant type; *P < 0.05; **P < 0.01. Table S1. The 29 PI3K pathway genes used to define samples as PI3K pathway mutation or wild groups. [file 12957_2023_2938_MOESM1_ESM.zip › Figure S3.tif]
